# Eco-epidemiological analysis of dengue infection during an outbreak of dengue fever, India

**DOI:** 10.1186/1743-422X-2-32

**Published:** 2005-04-14

**Authors:** Anita Chakravarti, Rajni Kumaria

**Affiliations:** 1 Department of Microbiology, Maulana Azad Medical College, Associated Lok Nayak Hospital, Bahadur Shah Zafar Marg New Delhi-110002, India

**Keywords:** Dengue Infection, Dengue fever, India, Rainfall, Temperature, Relative humidity

## Abstract

**Background:**

This study was designed to find out a relationship of dengue infection with climatic factors such as rainfall, temperature and relative humidity during the dengue fever epidemic in the year 2003. Blood samples were collected from 1550 patients experiencing a febrile illness clinically consistent with dengue infection. Serological confirmation of Dengue Infection was done using Dengue Duo IgM and IgG Rapid Strip test (Pan Bio, Australia), which detected dengue-specific antibodies. Monthly data of total rainfall, temperature and relative humidity for the year 2003 was obtained from Meteorological Department of Delhi, New Delhi and retrospectively analyzed.

**Results:**

Out of 1550 suspected cases, 893 cases (57.36%) were confirmed as serologically positive. The difference between numbers of serologically positive cases during different months was significant (p < 0.05). Larger proportions of serologically positive cases were observed among adults. Outbreak coincided mainly with the post monsoon period of subnormal rainfall. The difference between serologically positive cases as compared to serologically negative ones in post monsoon period was significantly higher (p < 0.001). The difference in the rainfall and temperature between three seasonal periods was significant (p < 0.05).

**Conclusion:**

This prospective study highlighted rain, temperature and relative humidity as the major and important climatic factors, which could alone or collectively be responsible for an outbreak. More studies in this regard could further reveal the correlation between the climatic changes and dengue outbreaks, which would help in making the strategies and plans to forecast any outbreak in future well in advance.

## Background

Dengue infection (DI) is amongst the most important emerging viral diseases transmitted by mosquitoes to humans, in terms of both illness and death [1]. The worldwide large-scale reappearance of dengue for the past few decades has turned this disease into a serious public health problem, especially in the tropical and subtropical countries [2-4]. It is estimated that 52% of the global population are at the risk of contracting Dengue fever (DF) or dengue hemorrhagic fever (DHF) lives in the South East Asian Region. Although all the four serotypes have been circulating in this region, ecological and climatic factors are reported to influence the seasonal prevalence of the dengue vector, Aedes aegypti, on the basis of which countries in this region are divided in to four zones with different DF/DHF transmission potential [5]. In most of the countries, dengue epidemics are reported to occur, during the warm, humid and rainy seasons, which favor abundant mosquito growth and shorten the extrinsic incubation period as well [6-9].

DF has been known to be endemic in India for over two centuries as a benign and self-limited disease. In recent years, the disease has changed its course manifesting in the severe form as DHF, with increasing frequencies [10] Delhi City (India) is home to more than 13 million people and is endemic for DI [11]. Overpopulation has consequently led to poor sanitary conditions and water logging at various places. A major epidemic of DHF from Delhi was last reported in the year 1996 after which DI became a notifiable disease and a number of policies were formulated to bring the DI as well as its vector under control. The retrospective studies, one conducted by us during the period, 1997–2001 and another by National Institute of Communicable Diseases (NICD), New Delhi during the year 1997, have observed a decline in the number of cases having either DF or DHF in the following years [12,13]. Although, the vector mainly responsible for the spread of DI is present all the year around in Delhi, studies on the relative prevalence and distribution have shown the highest *A. aegypti *larval indices during the monsoon and post monsoon period [13-15].

In the year 2003, India had experienced one of the wettest monsoons in 25 years, which led to a spate of mosquito growth creating an alarming situation of mosquito borne diseases in many states. Delhi experienced an outbreak of DF this year, after 6 years of silence. Studies conducted in the countries like Brazil, Indonesia and Venezuela, where DI is present either in epidemic or endemic form have suggested a correlation between weather and pattern of DI. Rain, temperature and relative humidity are suggested as important factors attributing towards the growth and dispersion of this vector and potential of dengue outbreaks [2-4]. Since limited data is available on the association of climatic conditions and the pattern of DI from this geographical region, this study was conducted to find out the relationship of dengue infection with climatic factors such as the rainfall, temperature and relative humidity during the dengue outbreak in the year 2003.

## Results

### Seropositivity

**Figure 1:**
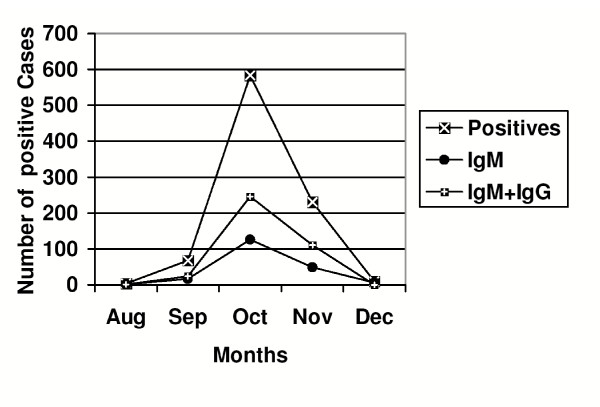
Month wise distribution of primary and secondary serologically positive cases during the outbreak period in the year 2003.

All blood smears microscopically screened for malarial parasite were found to be negative. Analytical interpretations presented in this study were based upon instructions mentioned in the Pan Bio Rapid Strip Test procedure manual. During the outbreak period, blood samples were collected from 1550 patients experiencing a febrile illness clinically consistent with DI over the period of one year from January to December 2003. Eight hundred ninety three cases (57.36%) were confirmed as serologically positive, out of which 199 (22.28%) cases were positive for dengue-specific IgM antibodies indicating primary infection and 381 (42. 67%) cases were positive for both dengue-specific IgM and IgG antibodies indicating secondary infection (Figure 1). IgG antibodies alone were also detected in 313 (35.05%) cases and these cases were presumed to be either suspected secondary dengue infection as IgG positivity alone could also be due to cross reactivity with other flaviviruses. The difference between numbers of serologically positive cases reported during different months was significant (p < 0.05).

DI is observed to be a seasonal disease in Delhi. According to intensity of rainfall, weather data was divided in three periods, namely; pre monsoon period: from February- May, monsoon period: from June – September and post monsoon period: from October – January. Few cases clinically suspected of dengue infection in the pre monsoon period were later found to be serologically negative for dengue-specific antibodies. During the monsoon period, only 3 cases (0.34%) were confirmed serologically positive in the month of August, and 68 cases (7.6%) in the September. Dengue-specific antibody positive cases were mainly reported during the post monsoon period with maximum number of cases 583 (65.3%) cases reported during the month of October followed by 230 (25.76%) cases in the November (Table 1). The difference between numbers of serologically positive cases as compared to serologically negative ones in post monsoon period was significantly higher (p < 0.001), than during the remaining period with 92% of total annual cases reported during this period.

**Table 1:** Month wise distribution of clinically diagnosed and serologically positive cases amongst primary and secondary cases during the DF outbreak, 2003

Month	Total Suspected cases	Serologically Positive cases (%)	Primary infection (IgM Positivity)	Secondary infection (IgM+ IgG Positivity)	Suspected secondary infection (IgG Positivity)
August	12	3 (0.34%)	1 (0.5%)	1 (0.26%)	1 (0.32%)
September	157	68 (7.6%)	17 (8.6%)	24 (6.3%)	27 (8.6%)
October	982	583 (65.3%)	126 (63.3%)	246 (64.57%)	211 (67.4%)
November	362	230 (25.76%)	49 (24.6%)	110 (28.87%)	71 (22.68%)
December	37	9 (1%)	6 (3%)	0 (0%)	3 (1%)
Total	1550	893 (57.36%)	199 (22.28%)	381 (42.67%)	313 (35.05%)

### Distribution by age

Out of 893 serologically positive cases, 687 cases belonged to the adult's age group (> 12 years) and 206 cases to pediatric age group (≤ 12 years) in this study. Larger proportions of serologically positive cases were observed among adults, with a positive prevalence of 56.4% among children and 58% among adults, distribution was however, not significantly different when compared with pediatric age group (p > 0.05). The difference between numbers of serologically positive cases among adult and pediatric group in post monsoon period as compared to the rest of the season was also not significant (p > 0.05) (Table 2).

**Table 2:** Month wise distribution of serologically positive cases amongst children and adults during the DF outbreak, 2003

Month	Dengue-specific Antibody Positive cases		
	Total	Children (Positivity %)	Adults (Positivity %)

August	3	0	3 (25%)
September	68	18 (48.6%)	50 (41.7%)
October	583	133 (69.6%)	450 (57%)
November	230	54 (44.3%)	176 (83.8%)
December	9	1 (11.1%)	8 (28.6%)
Total	893	206 (56.4%)	687 (58%)

### Climatic influence

**Figure 2:**
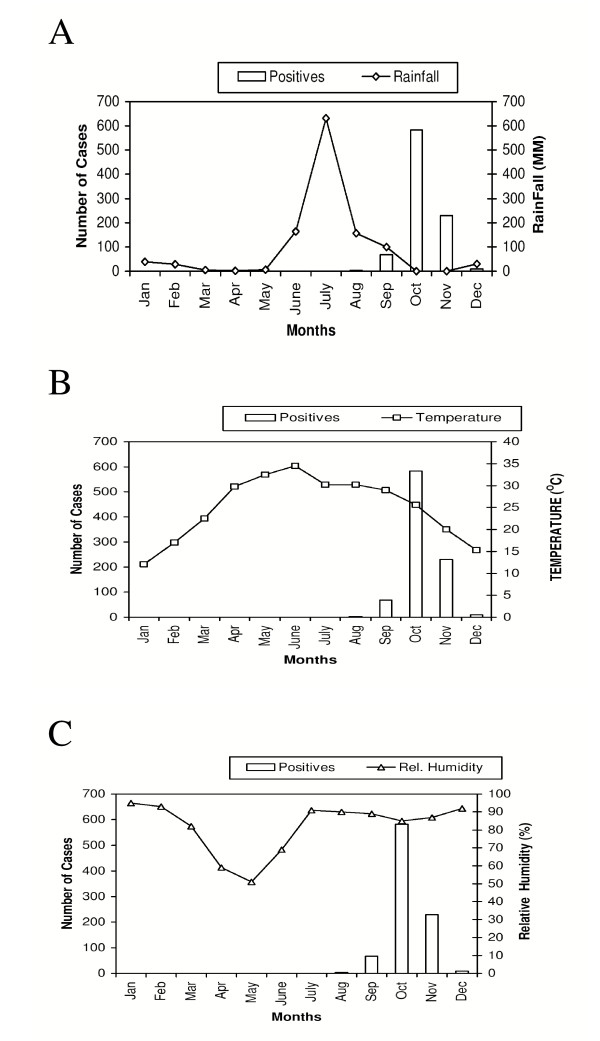
**a**: Month wise distribution of serologically positive cases of dengue fever /dengue hemorrhagic fever and rainfall in Delhi for the year 2003 **b: **Month wise distribution of serologically positive cases of dengue fever /dengue hemorrhagic fever and temperature in Delhi for the year 2003 **c**: Month wise distribution of serologically positive cases of dengue fever /dengue hemorrhagic fever and relative humidity in Delhi for the year 2003

Fig. 2a indicates that outbreak coincided mainly with the post monsoon period of subnormal rainfall (Cumulative rainfall = 30.3 mm) from October to December 2003 and was followed by relatively heavy rainfall during the monsoon period; from June to September 2003. The difference in the rainfall and temperature between three seasonal periods was found to be significant (p < 0.05) (Fig. 2a &2b). Mean ambient temperature was 25.4°C during the pre monsoon period, which increased to 30.9°C during the monsoon period; the period preceding the outbreak and decreased to 20.3°C (Mean temperature from October to December) in the actual outbreak months during the post monsoon period. The difference between relative humidity during the three periods was not significant. The mean relative humidity was 71.2% during the pre monsoon period. It increased during the monsoon period to 85% and increased further during the post monsoon period to 90% (Fig. 2c).

## Discussion

In the year 2003, India had experienced one of the wettest monsoons in 25 years, which led to a spate of mosquito growth creating an alarming situation of mosquito borne diseases in Delhi and many other states [16]. As a consequence to this unusually heavy rain, an outbreak of dengue fever was once again reported from Delhi after a silence of six long years. Most of vector borne diseases exhibit a distinctive seasonal pattern and climatic factors such as rainfall, temperature and other weather variables affect in many ways both the vector and the pathogen they transmit [17]. Worldwide studies have proposed that ecological and climatic factors influence the seasonal prevalence of both the *A. aegypti *and dengue virus [2-4]. The vector mainly responsible for the spread of DI is present at the basal level all the year around in Delhi, however, studies on the relative prevalence and distribution have shown the highest *A. aegypti *larval indices during the monsoon and post monsoon period [13-15]. Since limited data is available on the affect of climatic factors on the pattern of DI, this study was planned to carry out the month wise detailed analysis of three important climatic factors such as rainfall, temperature and relative humidity on the pattern of DI.

Observations on the seasonality were based on a single year's data as the intensity of sampling was at its maximum during this outbreak period. The outbreak coincided mainly with the post monsoon period of subnormal rainfall, which was followed, by relatively heavy rainfall during the monsoon period; from June to September 2003. The difference in the total rainfall and temperature during three seasonal periods was found to be statistically significant (p < 0.05). Monthly weather data showed that temperature variations were more amongst different months during the pre monsoon and post monsoon period as compared to the monsoon period. Even though, the monsoon season began in mid- June, there was no respite from the heat as there was not much difference in the temperature during the last month of pre monsoon; May and beginning of monsoon in the June. Unusual heavy rainfall subsequently led to decrease in temperature during the later part of monsoon period. The temperature showed a decline and remained almost constant during the months of July and August (30.2°C), continuous heavy rainfall subsequently led to further decrease in the temperature during the month of September to 29°C. Relative humidity increased during the rainy season and remained high for several weeks. An in-depth analysis of these three factors thus led to a proposal that optimum temperature with high relative humidity and abundant stocks of fresh water reservoirs generated due to rain, developed optimum conditions conducive for mass breeding and propagation of vector and transmission of the virus.

Our study was in tune with a previous study by NICD of seasonal variations and breeding pattern of *A. aegypti *in Delhi, which showed that there are two types of breeding foci, namely; primary and secondary breeding foci. Primary breeding foci served as mother foci during the pre monsoon period. *A. aegypti *larvae spread to secondary foci like discarded tyres, desert coolers etc., which collect fresh water during the monsoon period [14]. This study supported the proposal that all the three climatic factors studied could be playing an important role in creating the conducive condition required for breeding and propagation of this vector, the basal level of which is present all round the year. This prospective study therefore highlighted the major important factors, which could alone or collectively be responsible for an outbreak.

In our study, the largest proportion of serologically positive cases was recorded in the post monsoon period, which is in agreement with our previous study [12]. Our findings were in coordination with study by other groups from this geographical region [13-15]. The seasonal occurrence of positive cases has shown that post monsoon period is the most affected period in Bangladesh as well [18]. However, a retrospective study from Myanmar during 1996–2001 reported the maximum cases of dengue during the monsoon period [19]. Study by group of Rebelo from Brazil has also emphasized the importance of season. They have observed that dengue cases were higher during rainy season showing the importance of rain in forming prime breeding sites for *A. aegypti *thus spread of DI [20]. Study of eco-epidemiological factors by Barrera et al [21] showed that DF has a positive correlation with the relative humidity and negative relation with evaporation rate. Peaks of dengue cases were observed to be near concurrent with rain peaks in this study from Venezuela showing a significant correlation of intensity of DI with the amount of rain [21]. In this study we have observed that temperature tends to decrease towards the end of monsoon period, specially remains moreover constant during the later months of rainy season. India and Bangladesh fall in the deciduous, dry and wet climatic zone. The temperature remains high during the pre monsoon period. It is continuous rain pour for a couple of days that brings down the temperature during the monsoon period, which may also be responsible for an increase in the relative humidity and decrease in the evaporation rate thus maintaining secondary reservoirs containing rain water. More studies are needed to establish the relationship between the climatic changes and dengue outbreaks, which would help in formulating the strategies and plans to forecast any outbreak in future, well in advance.

Very little dengue is found in adults in Thailand, presumably because people acquire complete protective immunity after multiple DI as children [1], as DI is highly endemic in Thailand [22]. On the other hand, DI especially DHF is an emerging disease in India; probably this may be the reason that people of all the age are found to be sensitive to infection in our study. Even though more adults were reported of having anti dengue antibodies, the difference in the number of positive cases was not significant as compared to pediatric age group.

The severity of this outbreak was lesser as compared to the DHF epidemic that occurred in year 1996 caused by the serotype Den-2 [23]. Serotype Den-2 is reported to be the one mainly associated with DHF, the more severe form of the disease [24,25]. More studies in this regard can further elucidate correlation of serotypes with severity of disease from this geographical region.

## Conclusion

This prospective study highlighted rain, temperature and relative humidity as the major and important climatic factors, which could alone or collectively be responsible for an outbreak. More studies in this regard could further reveal the correlation between the climatic changes and dengue outbreaks, which would help in making the strategies and plans to forecast any outbreak in future well in advance.

## Materials and methods

### Study design, population and sample size

The present study was conducted retrospectively for a period of one year during the recent outbreak of dengue fever in Delhi in the year 2003. The study population comprised individuals of all age groups, attending the outpatient and inpatient departments of Lok Nayak Hospital, a tertiary care hospital in Delhi. Blood samples were collected from 1550 patients experiencing a febrile illness clinically consistent with dengue infection, selected according to the following inclusion and exclusion criteria.

### Case-inclusion criteria

A case was included if there was high fever with clinical symptoms suggestive of dengue infection as per WHO criteria [26].

### Case-exclusion criteria

A case was excluded, if routine laboratory testing suggested bacterial or any viral infection other than dengue infection or any other disease [26].

### Microscopy for malaria identification

Venous blood was used for blood slide preparation for malaria parasite examination. Thick and thin blood films were prepared on the same slide, stained with Giemsa and examined for the presence of malaria parasite.

### Laboratory confirmation of dengue infection by serology

Dengue Duo IgM and IgG Rapid Strip test (Pan Bio, Australia) was used for the detection of dengue-specific antibodies. 1 μl of serum was mixed with 75 μl of buffer (supplied in the kit) and test strip was dipped in to the diluted serum. Results of the test were read after 30 minutes. Serum antibodies of the IgM or IgG class, when present bind to anti-human IgM or IgG immobilized in two lines across the test strip. Colloidal gold-labeled anti-dengue monoclonal antibodies form complexes with the dengue antigen that is captured by dengue specific IgM or IgG in the patient's serum. These complexes were visualized as pink/purple line(s). The presence of anti-dengue IgM antibodies alone indicated primary infection. In contrast, presence of anti-dengue IgG antibodies with or without IgM indicated secondary infection. (IgG antibodies alone was considered as suspected secondary infection as it could also be due to cross reactivity with other flaviviruses).

### Analysis of metrological data

Monthly details of total rainfall, temperature and relative humidity for all the months of the year, 2003 were obtained from Meteorological Department of Delhi, Mausum Bhawan, New Delhi and retrospectively analyzed in relation to total number of dengue cases. According to the intensity of the rainfall, weather data was divided in three periods namely; pre-monsoon period: from February- May, monsoon period: from June – September and post monsoon period: from October – January.

## Competing Interests

The author(s) declare that they have no competing interests.

## Authors' contributions

It is stated that both the authors 1) have made substantial contributions to conception and design, or acquisition of data, or analysis and interpretation of data; 2) have been involved in drafting the article or revising it critically for important intellectual content; and 3) have given final approval of the version to be published.
